# Disentangling evolutionary signals: conservation, specificity determining positions and coevolution. Implication for catalytic residue prediction

**DOI:** 10.1186/1471-2105-13-235

**Published:** 2012-09-14

**Authors:** Elin Teppa, Angela D Wilkins, Morten Nielsen, Cristina Marino Buslje

**Affiliations:** 1 Fundación Instituto Leloir, Avda. Patricias Argentinas 435, CABA, C1405BWE, Argentina; 2 Department of Molecular and Human Genetics, Baylor College of Medicine, Houston, Texas; 3 Center for Biological Sequence Analysis, Technical University of Denmark, Lyngby, Denmark; 4 Instituto de Investigaciones Biotecnológicas, Universidad de San Martín, San Martín, B 1650 HMP, Buenos Aires, Argentina

**Keywords:** Coevolution, Mutual information, Specificity determining position, Catalytic residues, Functional sites, Sequence analysis

## Abstract

**Background:**

A large panel of methods exists that aim to identify residues with critical impact on protein function based on evolutionary signals, sequence and structure information. However, it is not clear to what extent these different methods overlap, and if any of the methods have higher predictive potential compared to others when it comes to, in particular, the identification of catalytic residues (CR) in proteins. Using a large set of enzymatic protein families and measures based on different evolutionary signals, we sought to break up the different components of the information content within a multiple sequence alignment to investigate their predictive potential and degree of overlap.

**Results:**

Our results demonstrate that the different methods included in the benchmark in general can be divided into three groups with a limited mutual overlap. One group containing real-value Evolutionary Trace (rvET) methods and conservation, another containing mutual information (MI) methods, and the last containing methods designed explicitly for the identification of specificity determining positions (SDPs): integer-value Evolutionary Trace (ivET), SDPfox, and XDET. In terms of prediction of CR, we find using a proximity score integrating structural information (as the sum of the scores of residues located within a given distance of the residue in question) that only the methods from the first two groups displayed a reliable performance. Next, we investigated to what degree proximity scores for conservation, rvET and cumulative MI (cMI) provide complementary information capable of improving the performance for CR identification. We found that integrating conservation with proximity scores for rvET and cMI achieved the highest performance. The proximity conservation score contained no complementary information when integrated with proximity rvET. Moreover, the signal from rvET provided only a limited gain in predictive performance when integrated with mutual information and conservation proximity scores. Combined, these observations demonstrate that the rvET and cMI scores add complementary information to the prediction system.

**Conclusions:**

This work contributes to the understanding of the different signals of evolution and also shows that it is possible to improve the detection of catalytic residues by integrating structural and higher order sequence evolutionary information with sequence conservation.

## Background

A number of methods have been developed to predict functionally important sites in protein families based on sequence and structure information. The importance of a particular residue in a protein can be due to many different factors, including structural stability, protein-protein interaction, protein-DNA/RNA interaction, ligand binding site and maintenance of protein functions.

In most cases, it is difficult to assign a particular function to a particular residue or group of residues, as function is determined by a subtle interplay between multiple residues and mutation to any of them might impact the protein function and/or structure. In some cases however, the association between a particular residue and a protein function can be readily recognized. One such example being catalytic residues, where large data set exist defining residues within a given protein sequence linked to a given catalytic function [1].

**Figure 1:**
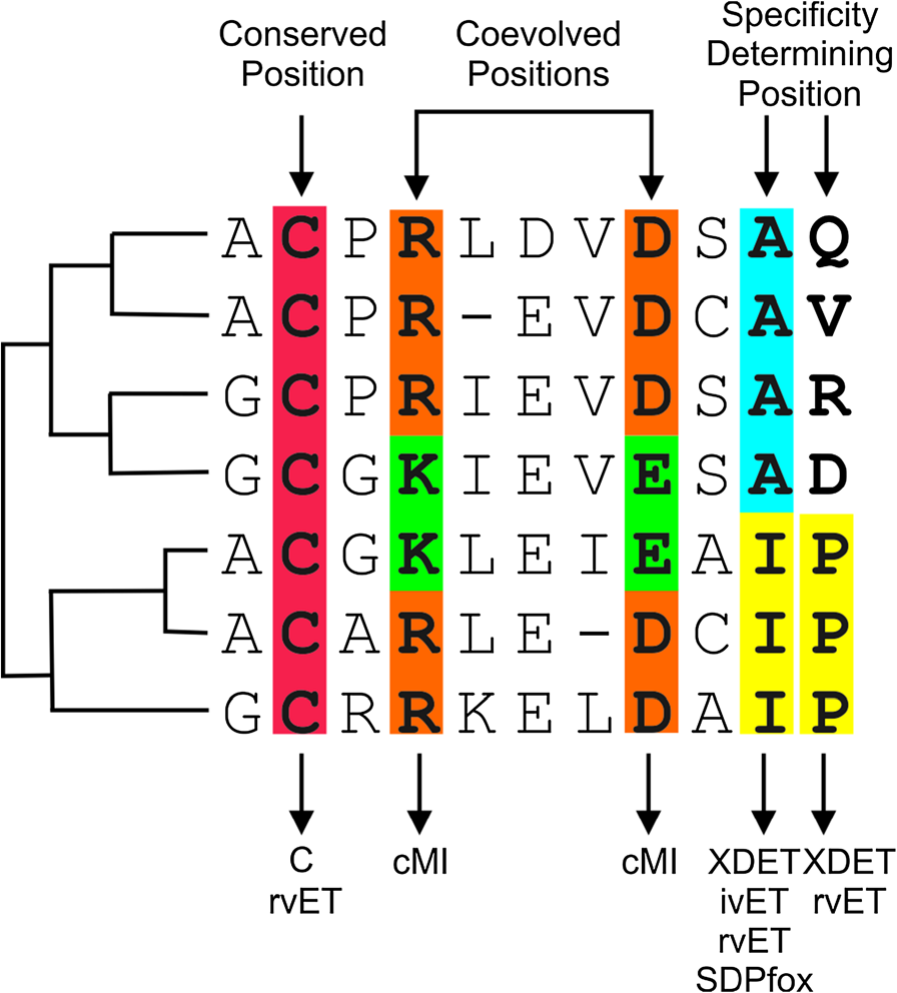
**Different column patterns in an MSA.** Schematic representation of an MSA and its phylogenetic tree (left). Conserved position is highlighted in red, coevolved positions in green and orange and putative SDPs in yellow and blue. On the top are indicated the column pattern and on the bottom, the suitable method to detect each kind of position (C: conservation score; cMI: cumulative MI; ivET: integer value ET; rvET: real value ET; XDET and SDPfox are also indicated).

Most methods developed to predict functionally important sites in protein families rely on some signal related to protein evolution. Three clear signals of evolution are: conservation, conservation within specific groups of sequences sharing a common function, and coevolution between residues (see Figure 1). Conservation is straightforward to calculate and interpret. A change in a conserved position (even when proteins are highly diverse) should have a deleterious effect on the protein function. Specificity determining positions (SDPs) are those positions within multiple sequence alignments (MSAs) that are conserved within groups of proteins that perform the same function (specificity groups) and varying between groups with different functions/specificities. These sites generally determine protein specificity either by binding specific substrate/inhibitor or through interaction with other protein [2-4].

The degree of co-evolution between pairs of residues is commonly estimated using a measure of mutual information (MI). If two residues share high signal of mutual information, the two residues most likely are co-evolving, meaning that in order to maintain a given protein function a mutation of one residue is linked to a specific compensatory mutation of the other residue.

Several methods to predict specificity-determining positions have been developed. Many of these require a previous classification of the proteins into functional groups [3,5,6], which is a problematic limitation since the specificity of a given protein is unavailable in the great majority of cases and is non-trivial to calculate and validate. To overcome this problem, methods have been developed that group the sequences in a MSA upon certain ad-hoc criteria [7,8]. As an example, Capra and Singh [9] addressed the classification problem using a combination of Pfam, EC numbers and sequence similarity. There are also methods where the clustering is based on sequence similarity alone [10] or Bayesian statistics [11]. Many of these methods approximate the classification of sequences using phylogeny [12-14] or a combination of phylogenetic information and entropies analysis [15]. Other methods rank residues by their relative importance in the MSA [12,13,16-18]. These approaches differ in design, but all look for specific patterns of amino acids conservation as indicators of likely functional importance.

Finally, inter-relationship between two or more positions (estimated using mutual information) can contribute a different type of biological information related to protein function and functional importance of specific residues. We have earlier introduced a cumulative mutual information concept (cMI) that measures the degree of shared mutual information of a given residue and the proximity mutual information (p(MI)) which measures the amount of shared mutual information in the proximity of a given residue [16]. In a large benchmark data set of enzymatic protein families, we showed that whereas identification of catalytic residues (CR) is strongly guided by sequence conservation, mutual information (or coevolution) provides an additional and complementary signal that significantly improves the predictive power.

A large panel of methods thus exists aiming at identifying residues with critical impact on protein functionally relying on measures of information content extracted from multiple sequence alignments. However, it is not clear to what extent the predictive power of the different methods overlap, and if any of the methods have higher predictive potential compared to others when it comes to the identification of a particular type of functional important sites. Here, we aim at addressing this question by comparing the ability to identify CR in enzymatic proteins of different information-based methods. Although CR clearly do not constitute the sole test-case to perform such an investigation we have chosen this test-case due to the large data sets of unambiguous annotations of functionally important residues available.

Using this test-case of CR identification, we seek to decompose and compare the predictive signal of a series of unsupervised (i.e. methods that do not require prior functional clustering) information-based predictions method. The analysis includes on the one hand, methods aim at ranking residues by their functional importance using i) conservation; ii) mutual information [16] and iii) evolutionary trace real value (rvET) that incorporates evolutionary and entropic information from multiple sequence alignments [13]. On the other hand, we include methods aimed at detection of specificity positions i.e. i) the evolutionary trace integer value (ivET) score that represents conservation within groups in a qualitative manner [12]; ii) SDPfox [10] that predicts SDPs in a phylogeny-independent manner and iii) XDET [19] that is based on the comparison of the mutational behavior of a position with the mutational behavior of the full-length protein MSA, by directly comparing the corresponding distance matrices.

Comparing these methods will allow us to break up the different components of information content included in a MSA, investigate to what degree they overlap and estimate their predictive potential for the identification of active site residues in catalytic proteins.

## Results and discussion

The analysis is based on a set of 424 enzymatic Pfam families earlier described by Marino Buslje (2010) [16] (for details see Methods). In short, each family is characterized by a (MSA) taken from Pfam [20], has an annotated set of CR taken from the CSA database [1] and by having a known three-dimensional structure for at least one of its members. Given this data set, we calculated measures related to evolution for the different methods included in the benchmark, and next analyzed the overlap/correlation between these measures and their predictive potential for identification of CR in proteins.

Although all the methods are intended to identify functionally important sites within protein families, they can be divided into two major groups: the methods that rank positions in the MSA according to their relative functional importance within the protein family, no matter what this importance might be due to. In this category falls the cumulative mutual information (cMI), real-value evolutionary trace (rvET) and sequence conservation (C). The other group consists of methods intended to predict specificity determining positions in a family of proteins and includes XDET, ivET and SDPfox.

### Concordance of the different predictions methods

**Figure 2:**
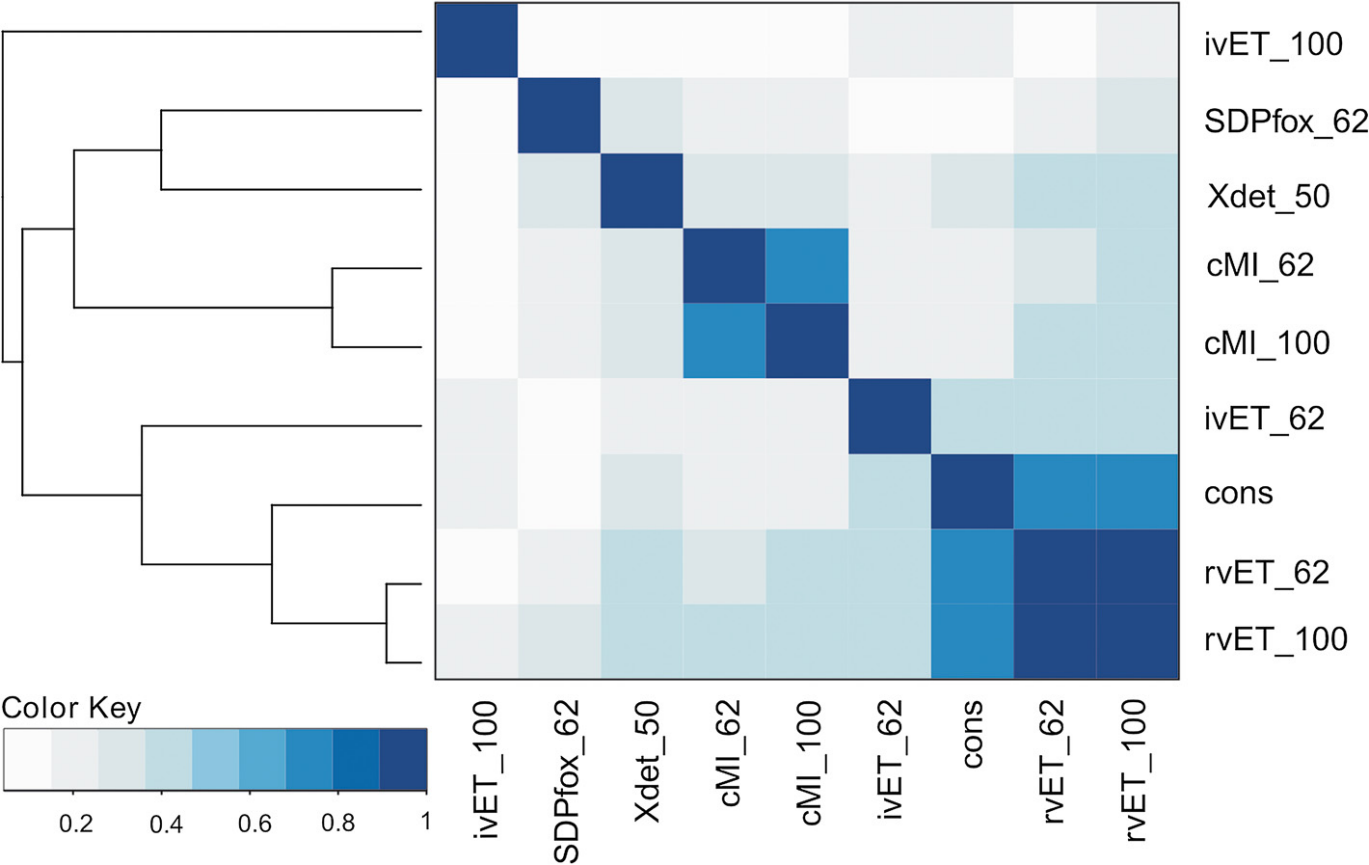
**Heat map representation of the Spearman rank correlation coefficient between methods.** cMI: cumulative MI, ivET: integer value evolutionary trace, rvET: real value evolutionary trace, cons: conservation. Numbers following the methods name (100, 62 and 50) indicate the redundancy of the sequences in the MSA (100, 62 and 50% redundancy reduced). The dendrogram indicates the distance between methods. Correlation colour key goes from white (0, no correlation) to blue (1, perfect correlation). All correlations are statistically different from zero (T-test, p-value threshold of 0.05).

To determine the influence data redundancy might have on the prediction scores for the different methods, we measured the correlation between scores calculated on the MSAs as retrieved from Pfam (MSA100) and on a set of sequence redundancy reduced MSAs (for details see materials and methods). If a given method is insensitive to sequence redundancy, the scores produced from the different MSAs should be highly correlated. This is true for cMI (Spearman’s rank correlation coefficient, SCC = 0.76) and rvET (SCC = 0.93). However, for ivET we found only a weak correlation between the scores obtained using the two data sets (SCC = 0.21) indicating that data redundancy for this method strongly impacts the predictive output (see Figure 2, Additional file 1 Table S1 and Additional file 2 Figure S1).

Methods for prediction of SDPs aim at estimating a score that correlates with the functional importance of a given residue in terms of protein specificity. Another critical question to analysis is therefore the degree of concordance between different prediction methods aiming at identifying SDPs. From Figure 2, it is clear that the methods for SDP identification (ivET, SDPfox and XDET) show limited mutual overlap. The correlations values are low for all comparisons, with the highest value of 0.34 being between SDPfox and XDET.

Next, we investigated to what degree the information extracted by the methods developed for detection of SDPs (ivET, SDPfox, XDET) overlapped with the information signal of cMI, which points out positions with a high degree of shared mutual information. We found that cMI has a low overlap with all these methods (SCC < 0.28 for every comparison, see Figure 2 and Additional file 1 Table S1).

We next analyzed the correlation between methods aimed to rank the residues by functional importance (rvET, cMI and conservation). As expected, conservation was strongly correlated with rvET [13] for both the MSA100 and MSA62 (redundancy reduction at 62%) data sets (SCC > 0.7, in both cases). cMI was found to be weakly correlated with conservation (SCC 0.16 for both MSA100 and 62), and finally the overlap between the rvET and cMI methods was moderately weak with a maximal correlation of 0.41.

The above results demonstrate that the different methods included in the benchmark in general can be divided into major groups with a limited mutual overlap. One group containing the methods with a signal highly correlated to sequence conservation (rvET, conservation and ivET when was evaluated on redundancy reduced data). Another group containing the methods which signal is derived from mutual information (cMI). The methods designed explicitly for the identification of SDPs (SDPfox and XDET) have low correlation to any other method included in the benchmark. The ivET method evaluated on the MSA100 data set (ivET100) appears as an outlier in this analysis and does not show overlap with any other method. The results hence in general underline that the overlap between the different methods in most cases is limited, suggesting that high ranked cMI and SDPs do not necessarily form the same group of residues. Also, it is noticeable that methods aimed to detect the same kind of positions as SDPs (ivET, SDPfox, and XDET) display rather low mutual concordance in prediction scores.

### Proximity summed information measures for predicting catalytic residues

We have earlier demonstrated that CR are characterized by a structural proximity with high mutual information, i.e. that residues within a certain distance threshold of CR are rich in shared MI [16]. To investigate if similar observations can be made for the other information measures included in the benchmark, we calculated a proximity measure of each method and investigated to what degree this measure contributed to the identification of CR. For each residue, we calculated the proximity score as the sum of the scores of the residues located within a certain distance from the residue in question (see equation 1)

(1) $$pMI_{i}=\sum_{j,d_{\mathrm{ij}}<t}cMI_{j}$$

where the sum is over all residues j in the given protein within a distance d_ij_ < t from the residue i, where d_ij_ is the shortest distance between any pair of heavy atoms of two residues i and j, cMI_j_ is the cumulative mutual information score of residue j, and t a distance threshold. Those measures are designated with a “p” preceding the name of each method, i.e.: p(rvET) for proximity rvET, p(ivET) for proximity ivET, p(C) for proximity C, and p(MI) for proximity cMI. The threshold distance t was optimized for each prediction method.

Table 1 gives the results of the calculation, and shows that all methods with the exception of p(ivET) evaluated on the MSA100 data set, SDPfox and XDET could be used as reasonable predictors of CR (AUC > 0.8 for all the methods). Note that we also here observe a large difference in the performance of the ivET method when evaluated on the different sets of MSAs. The results in Table 1 shows that all high performing methods (AUC > 0.8) have a optimal proximity distance threshold between 5 and 7 Å. Only for the SDPs prediction methods which all have poor predictive performance is the distance threshold larger.

**Table 1:** Performance and optimal distance threshold of the proximity measures for detecting catalytic residues

**Method**	**AUC average**	**Distance cutoff (Å)**
p(SDPfox62)	0.703	12
p(XDET50)	0.736	8
p(ivET62)	0.835	7
p(ivET100)	0.640	7
p(rvET62)	0.878	5
p(rvET100)	0.875	7
p(MI62)	0.823	7
p(MI100)	0.833	7
p(C)	0.854	5

The number of protein families included in SDPfox is 289, 298 in XDET and 424 in all other methods. “p” before the method’s name denotes “proximity”. The number following the method’s name denotes the MSA data set on which the method was evaluated (ie: 50 = MSA50). The optimal distance cut-off for the proximity sum was found using a grid-search as described in Methods.

We can next investigate to what degree the performance of the different methods is statistically different. In doing this, we obtain the following rank of the methods:

$$\begin{array}{l} p\left(\text{rvET}62\right)\approx p\left(\text{rvET}100\right)\approx p\left(C\right)>p\left(\text{ivET}62\right)\\ \;\approx p\left(\text{MI}100\right)\approx p\left(\text{MI}62\right)>p\left(\text{XDET}50\right)\\ \;\approx p\left(\text{SDPfox}62\right)>p\left(\text{ivET}100\right) \end{array}$$

Here a “≈” means that the preceding value is higher but not statistically different and “>” means significantly higher, where statistical tests were conducted as binomial tests excluding ties using a p-value threshold of 0.05. The different methods hence fall in three different groups a) p(rvET62), p(rvET100) and p(C), b) p(ivET62), p(MI100) and p(MI62), c) p(XDET50) and p(SDPfox62), with p(ivET100) as an outlier.

### Combined catalytic likeliness Score (Cls) with the best performing distance threshold for each method and optimizing the weight for each term

We have demonstrated in a previous work how the p(C) and p(MI) scores when integrated with conservation enhance the predictive performance for identification of CR [16]. Here, we aimed at demonstrating to what degree this observation is maintained when integrating the other methods included in the benchmark with the conservation score. In this way, we can in a simple manner investigate to what degree each method adds complementary information to the final prediction model. We defined different combined models by adding one or more proximity scores to the conservation score. For each Pfam family, the additional feature was normalized so that the values fell in the range [0–1] (for details see Methods). We included in this benchmark p(MI62) (previously used for CR detection [16]), the best performing ET method p(rvET62), and p(C).

Table 2 gives the performance values in terms of the AUC (area under the ROC curve) and AUC0.1 (area under the ROC curve integrated up to a false positive rate of 0.1) and optimal relative weights (estimated using 5 fold cross-validation) for the different models. The 0.2·C + 0.8·p(C) row hence gives the optimal performance for the model defined as a combination of conservation (C) and the proximity sum of conservation (p(C)), and states that the optimal relative weight of the two terms is 0.2 on conservation and 0.8 on p(C), respectively. In the table, a weight equal to 0 indicates that a given score did not contribute to the performance of the model.

**Table 2:** Performance of different methods in terms of the AUC

**Method**	**AUC**	**AUC0.1**
C	0.881	0.491
0.2 C + 0.8 p(C)	0.898	0.553
0.15 C + 0.85 p(rvET62)	0.913	0.567
0.25 C + 0.75 p(MI62)	0.912	0.555
0.15 C + 0.0 p(C) + 0.85 p(rvET62)	0.913	0.567
0.15 C + 0.3 p(C) + 0.55 p(MI62)	0.916	0.571
0.15 C + 0.0 p(C) + 0.45 p(rvET62) + 0.4 p(MI62)	0.921	0.586

Methods give the optimal combined model including conservation and the different proximity scores. The relative weights were determined using fivefold cross validation as described in the text. AUC and AUC0.1 are the average performance values over the 424 protein families.

Several observations can be made from these results. First of all, it is clear that all proximity scores contain complementary information that when combined with conservation (C) leads to an improved predictive performance (all models C + p(XX), where XX equals C, rvET62, or MI62 significantly outperform the model based on conservation only, p < 0.05 one-tailed binomial test excluding ties). Also, it is striking to observe that the relative weight on the p(C) score in all models including p(rvET) is zero. This strongly suggests that the high performance of the p(rvET) method shown in Table 1 is driven by the signal of sequence conservation contained within the rvET score (as also suggested from the correlation analysis in Figure 2). The model C+p(C) +p(MI), achieved a higher performance than the corresponding model C + p(C) + p(rvET), the difference is however not statistically significant (p > 0.1, one-tailed binomial test excluding ties). Finally, the model C + p(rvET) + p(MI) integrating both the cMI and rvET proximity scores had the highest performance of all models included in the benchmark, and significantly outperformed all other models, except C + p(C) + p(MI) (p < 0.05 in all case, one-tailed binomial test excluding ties). In terms of specificity and precision, the C + p(rvET) +p(MI) method had average performance values of 0.88 and 0.25 respectively at a sensitivity level of 0.98 when evaluated on the 424 Pfam data sets. At a sensitivity level of 0.55 these values are altered to 0.96 and 0.45, respectively. For comparison, these values are reduced to 0.82/0.20 and 0.93/0.35, respectively, using the model defined from conservation only. Note, that these conclusions are maintained integrating multiple residue-specific information measures (conservation, rvET, and cMI) with the corresponding proximity scores. Doing this, we confirm that both the rvET and cMI measures carries complementary information, and that this complementarity is captured both at the per-residue and proximity level (data not shown).

Taken together, these observations demonstrate that the rvET and cMI scores capture distinct signals from the MSA and add complementary information to the prediction system.

## Conclusions

Many algorithms have been proposed for the identification of residues critical for protein function in general and protein specificity in particular. Here, we have compared a series of such methods in terms of both the concordance between their predictions and their ability to identify catalytic sites in proteins with enzymatic function. From our results, we find that the methods included in the benchmark can be divided in three groups with limited mutual overlap. One group consists of methods which predictive signal is strongly correlated to sequence conservation (rvET, and sequence conservation itself), one group consists of the methods whose predictive signal is derived from mutual information (cMI), and the last group consists of the methods developed for prediction of specificity determining positions (SDPfox, XDET and ivET).

Defining a proximity score for each method as suggested by [16] and benchmarking for the ability to identify CR, we find that only methods from the first two of the above three groups displayed a reliable predictive performance (mean AUC value above 0.8), indicating that the methods from the SDP group has limited value for the identification of residues critical for protein function. Comparing the different methods for prediction of specificity determining positions we found that they shared limited mutual overlap despite the fact that they are designed to capture a common functional signal.

Finally, we investigated to what degree the information signal of conservation, rvET and cMI methods (belonging to the two well-performing groups of methods) was complementary so that the combined signal could significantly improve the predictive capacity. Here, we found that the predictive performance could be significantly improved when combining conservation with the signal from the proximity scores of the different methods. The best performing method was found to consist of a combination of sequence conservation and proximity scores for both rvET and cMI. This finding confirms the notion that the rvET and cMI methods are distinct in nature, and that the two methods add informative and complementary information to the prediction system. The benchmark however also demonstrated that the gain in predictive performance of the rvET signal is limited and insignificant if combined with the conservation proximity scores.

It is critical to emphasize that the conclusions obtained in this work are strictly related to the identification of CR in enzymatic proteins. Albeit the different methods for predicting SDPs do not correlate strongly in our dataset, some have proven to be successful in the predictions in small size benchmark data sets with a limited number of sequences and few specificity groups [21-23]. Capra et. al (2008) [9] obtained reasonable results in predicting SDPs using as true positive, predicted instead of experimentally determined SDPs. Also, successful prediction results were obtained by Rodriguez GJ (2010) [24], demonstrating with experimental verification that they were able to predict, with rvET, residues responsible for the specificity between dopamine and serotonine ligands in bioamine receptors of the Class A G-Protein coupled receptors family.

What remains an unquestionable result from our analysis is that prediction scores for the different methods evaluated share a limited overlap, and in particular that the methods for SDP identification and the method based on mutual information capture a very distinct signal of evolutionary information.

In conclusion, we believe this work contributes to: i) a better understanding of the different signals of evolution of a protein; ii) in a highly quantitative manner characterize similarities and differences between different information measures captured within a multiple sequence alignment and iii) demonstrates that it is possible to significantly improve the ability to detect CR by integrating these different types of information measures.

## Methods

### Data set construction

The data set comprise 424 Pfam multiple sequence alignments (MSA) with CR annotation in Catalytic Site Atlas database (version 2.2.11, released August 2009) [1] earlier published by [16]. CSA provides catalytic site annotation for enzymes in the PDB. Catalytic residues are defined as those residues directly involved in some aspect of the reaction catalysed by an enzyme. Note, that the data is different from the original publication [16] due to parsing errors for 10 MSAs. We ended up with a dataset of 424 Pfam families which in turn include a total of 1328 CSA annotated catalytic residues. Each family has on average 3 CR (standard deviation 1.72) with a minimum of 1 and a maximum of 23 CR per family. The distribution of the number of sequences per families is shown in Additional file 3: Figure S2.

For each family at least one three-dimensional structure is known and this protein sequence was taken as a reference. When more than one PDB entry with catalytic site annotation was available for a given family, one reference PDB entry was selected following the criteria: highest sequence coverage of the Pfam MSA, the year of structure determination (preferably later than 2000) and resolution. Multiple sequence alignments were taken from Pfam [20] and pretreated by trimming deletions and insertions across the whole alignment so as to preserve the continuity of the reference sequence.

In order to investigate the effect of sequence redundancy on the different methods, we tested the performance of the MI and ET methods using the full multiple alignments (as retrieved form Pfam) as well as in a set of redundancy reduced alignments (reduced at 62% identity). SDPfox and XDET were tested only with a set of MSA 62% and 50% identity redundancy reduced respectively, due to their limitation in the number of input sequences allowed and the large runtime requirements (see below). The different benchmark data sets are named MSA100 (no redundancy reduction applied), MSA62 and MSA50 respectively. Redundancy reduced alignment were generated with T-Coffee software [25]. The complete data sets of MSAs for the 424 Pfam families, including catalytic site annotations is available at http://www.cbs.dtu.dk/suppl/immunology/CSA.

### SDP prediction software

SDPs predictions were performed with: a) Integer value ET (ivET) score that represents conservation within groups in a qualitative manner [12]; b) SDPfox method that predicts SDPs in a phylogeny-independent manner [10]. The software was downloaded from http://bioinf.fbb.msu.ru/SDPfoxWeb/main.jsp and run locally with default parameters. This method has a limitation on the number of specificity groups per family (between 2 and 200 specificity groups) and total length of the sequence (<500 residues), so the predictions for this method were hence made on the MSA62 data set; and c) XDET software is based on the comparison of the mutational behavior of a position with the mutational behavior of the whole alignment [19,26]. It furnishes two methods for detecting position related to functional specificity. Here, we used the mutational behavior (MB) method of XDET that does not use external arbitrary functional classification. Due to the high cost of the computer time of the method (the running time grows quadratic with the number of sequences) it was only possible to run XDET on the MSA50 data set. Source code for XDET was obtained from the authors.

### Methods of functionally important residues prediction

Prediction of functionally important residues was performed with the following methods: a) Sequence conservation, was calculated from the MSA100 as the Kullback–Leibler relative entropy [27] using an amino acids background frequency distribution obtained from the UniProt database (http://www.uniprot.org/); b) Mutual information was calculated in terms of the cumulative Mutual Information (cMI) score, that measures the degree of shared mutual information of a given residue [16]; c) Evolutionary Trace real value score (rvET) [24], which incorporates entropy as a quantitative measure of conservation giving a rank of positions by their relative importance.

### Predictive performance

The predictive performance in detecting CR using the proximity scores was evaluated in terms of the area under the ROC curve (AUC) per family. Annotated CR in the CSA were taken as the positive set, and all the other residues were assigned as negative. Both the full AUC value and the value integrated for specificities from 1 to 0.9 were included to capture the high specificity performance of the different measures [28]. The overall predictive performance was evaluated as a simple average of the per-family obtained AUC values. Parameters for each model were optimized using fivefold cross validation.

### Proximity summed scores

We calculated proximity scores for each method as a sum of the scores within a certain physical distance to the given amino acid. The distance between each pair of residues in the structure was calculated as the minimum distance between two heavy atoms. The optimal distance threshold for each proximity measure was found using a grid of 3, 4, 5, 6, 7, 8, 9, 10, 11 and 12 Å.

### Derived scores to predict catalytic residues

To integrate different score with sequence conservation, a combined score was defined as (equation 2)

(2) $$S=\left(1-w\right)\;C+wX$$

where w is a relative weight in the range [0–1]. For each protein family MSA, the additional feature was normalized so that the values fell in the range [0–1]. This single combination was made for X = {p(C), p(rvET62) and p(MI62)}. Note that for p(ET), the formula is $S=\left(1-w\right)\;C-w\;p\left(rvET62\right)$, as ET best rank is the smallest number.

When two features are added, the combined score was calculated as equation 3.

(3) $$S=\left(1-w_{1}-w_{2}\right)C+w_{1}\;p\left(C\right)+w_{2}\;X$$

Where $w_{1}$ and $w_{2}$ are relative weights both in the range [0–1], and $w_{1}$ +$w_{2}$< 1. Here the combination was made for X = {p(rvET62) and p(MI62)}. Also here the sign for the last term was negative when X = p(rvET62).

Finally, the complete combination of all methods was calculated as equation 4.

(4) $$\begin{array}{l} S=\left(1-w_{1}-w_{2}-w_{3}\right)\;C+w_{1}\;p\left(C\right)\\ \;-w_{2}\;p\left(rvET62\right)+w_{3}\;p\left(MI62\right) \end{array}$$

where $w_{1}$, $w_{2}$ and $w_{3}$ are relative weights in the range [0–1] and $|w_{1}+w_{2}+w_{3}<1$.

## Competing interests

The author(s) declare that they have no competing interests.

## Author’s contributions

ET: Contributed to the acquisition, analysis and interpretation of data, results discussion and drafting the manuscript. AW: acquisition of data, discussion and manuscript revision; MN and CMB: conceived the study, participated in its design and coordination, results analysis, discussions and writing of the manuscript. All authors read and approved the final manuscript.

## Supplementary Material

Additional file 1 **Table S1.** Spearman rank correlation between methods and their standard deviation. Click here for file

Additional file 2 **Figure S1.** Schematic representation of the sequence redundancy impact on ivET predictions. Click here for file

Additional file 3 **Figure S2.** Distribution of the number of Pfam families vs number of sequences per family. Click here for file
