# Supplementary material for: Disentangling evolutionary signals: conservation, specificity determining positions and coevolution. Implication for catalytic residue prediction
Source: BMC Bioinformatics. 2012 Sep 14;13:235. doi: 10.1186/1471-2105-13-235 (PMC3515339; doi:10.1186/1471-2105-13-235)
Supplement: Additional file 1 — Table S1. Spearman rank correlation between methods and their standard deviation. [file 1471-2105-13-235-S1.pdf]

Supplementary Table 1: Spearman rank correlation between methods and their standard deviation.

	SDPfox62	XDET50	ivET62	ivET100	cMI62	cMI100	rvET62	ivET100	cons
SDPfox62	1								
XDET50	0.34±0.2	1							
ivET62	0.10±0.13	0.22±0.13	1						
ivET100	0.07±0.14	0.08±0.13	0.21±0.21	1					
cMI62	0.16±0.26	0.26±0.21	0.17±0.18	0.04±0.16	1				
cMI100	0.16±0.27	0.28±0.21	0.18±0.19	0.05±0.18	0.76±0.15	1			
rvET62	0.23±0.16	0.38±0.15	0.41±0.24	0.14±0.26	0.36±0.23	0.38±0 .21	1		
rvET100	0.26±0.17	0.38±0.15	0.41±0.22	0.21±0.29	0.36±0.22	0.41±0 .20	0.93±0.10	1	
cons	0.05±0.12	0.31±0.16	0.44±0.17	0.17±0.22	0.16±0.22	0.16±0 .19	0.74±0.15	0.73±0.15	1
