# Supplementary material for: Disentangling evolutionary signals: conservation, specificity determining positions and coevolution. Implication for catalytic residue prediction
Source: BMC Bioinformatics. 2012 Sep 14;13:235. doi: 10.1186/1471-2105-13-235 (PMC3515339; doi:10.1186/1471-2105-13-235)
Supplement: Additional file 2 — Figure S1. Schematic representation of the sequence redundancy impact on ivET predictions. [file 1471-2105-13-235-S2.pdf]

Supplementary Figure 1:
Schematic representation of the sequence redundancy impact on ivET predictions

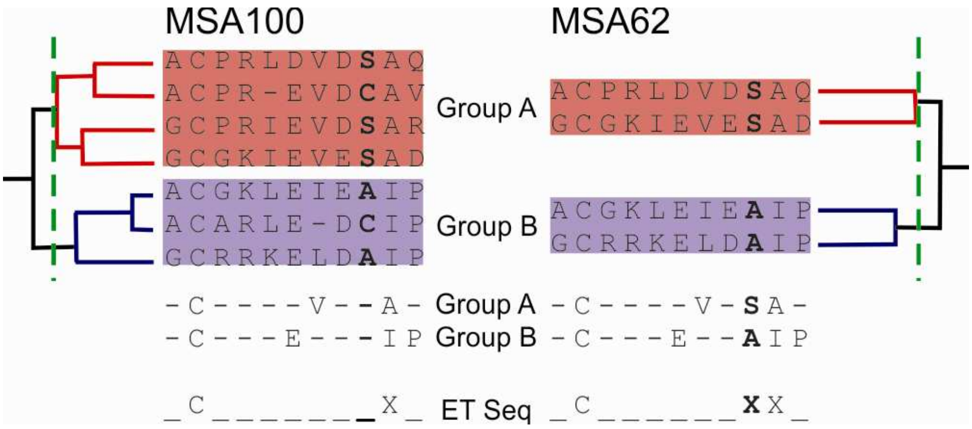

ET results on a hypothetical redundant MSA (left) and a redundancy reduced MSA (right). A possible partition of the phylogenetic tree (green dashed line) generating two groups is shown.

For each group a consensus sequence is constructed showing invariant positions by the amino acid and variable positions by "-". These consensus sequences are next compared to generate the Evolutionary Trace sequence (ET seq). Only positions with group-specific conservation (an invariant residue in group A and a different residue also invariant in group B) are predicted as SDP and showed with an X. The differences in prediction between the two alignments are highlighted in bold.
