# Supplementary material for: Disentangling evolutionary signals: conservation, specificity determining positions and coevolution. Implication for catalytic residue prediction
Source: BMC Bioinformatics. 2012 Sep 14;13:235. doi: 10.1186/1471-2105-13-235 (PMC3515339; doi:10.1186/1471-2105-13-235)
Supplement: Additional file 3 — Figure S2. Distribution of the number of Pfam families vs number of sequences per family. [file 1471-2105-13-235-S3.pdf]

Supplementary figure 1:
Distribution of the number of Pfam families vs number of sequences per family.

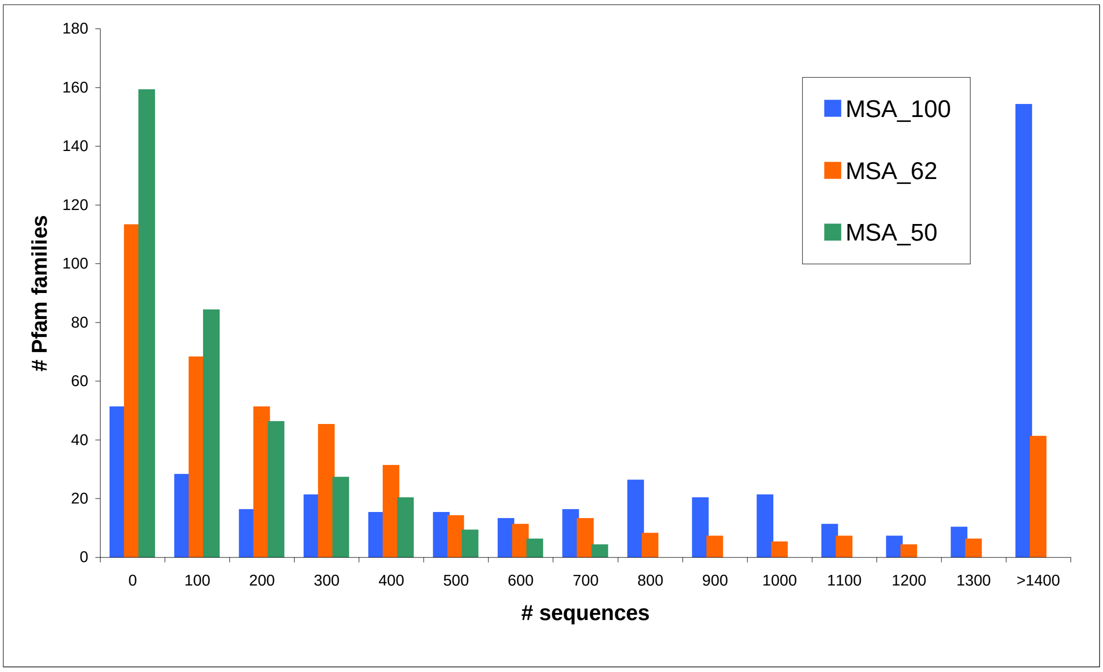

# of sequences	MSA_100	MSA_62	MSA_50
0	51	113	159
100	28	68	84
200	16	51	46
300	21	45	27
400	15	31	20
500	15	14	9
600	13	11	6
700	16	13	4
800	26	8	0
900	20	7	0
1000	21	5	0
1100	11	7	0
1200	7	4	0
1300	10	6	0
>1400	154	41	0
